# Experiences of Interpersonal Trauma Among Parents With Intellectual Disabilities: A Systematic Review

**DOI:** 10.1177/15248380221119237

**Published:** 2022-09-05

**Authors:** Mårten Hammarlund, Pehr Granqvist, Tommie Forslund

**Affiliations:** 1 Department of Psychology, Stockholm University, Sweden; 2 SUF Resource Center, Uppsala, Sweden

**Keywords:** intellectual disabilities, interpersonal trauma, risk factors, abuse, maltreatment, caregiving, child development

## Abstract

Research has suggested highly elevated levels of interpersonal trauma (IPT) among parents with intellectual disabilities (ID), and that such experiences may contribute to the caregiving and child developmental problems often seen in this population. Conflicting results have however been reported, and there is no systematic review on this matter. This study therefore systematically reviewed the empirical evidence concerning (a) prevalence of IPT among parents with ID, and links with (b) caregiving-relevant and (c) child developmental outcomes, in accordance with the PRISMA 2020 guidelines. Searches were conducted in MEDLINE, CINAHL, PsycINFO, and PTSDpubs. Peer-reviewed empirical articles reporting exposure to any form of systematically assessed IPT (unspecified IPT, physical, sexual, and emotional abuse, neglect, prolonged childhood separations from caregivers, witnessing abuse in the family) among parents with ID were included, yielding a final selection of 20 studies. Findings consistently indicated markedly elevated levels of IPT among parents with ID, with a majority (>50%) having experienced some form of IPT. Estimates for both unspecified and specific forms were typically higher than corresponding estimates in other groups at elevated risk, and than meta-analytical general population estimates in comparable countries. Findings regarding caregiving-relevant outcomes were mixed but indicated links with adverse outcomes, particularly regarding parental mental health. Reports pertaining to child developmental outcomes were scant and inconsistent. We highlight important limitations in the extant literature and provide directions for future research and clinical practice.

Parents with intellectual disabilities (ID; *IQ* < 70 and limitations in adaptive functioning; Schalock et al., 2021) and their children constitute some of society’s most vulnerable families. The parents are at heightened risk for encountering caregiving difficulties, including decreased basic childcare skills, problem-solving difficulties, and inconsistent responses to children’s signals (e.g., MacLean & Aunos, 2010). Children of parents with ID also have elevated risks for negative outcomes, including mental health problems (e.g., Neely-Barnes et al., 2014), behavioral problems (e.g., Cleaver & Nicholson, 2007), and developmental delays (e.g., Emerson & Brigham, 2014). Additionally, the families are at heightened risk for child protection involvement (e.g., McConnell et al., 2011), and for family separation due to out-of-home placements (e.g., McConnell et al., 2002).

It has commonly been assumed that the aforementioned risks originate in the parents’ ID per se (for a discussion, see McConnell & Llewellyn, 2002). As a result, parental ID has regularly been viewed as an indication of inadequate caregiving and a sufficient reason for revoking parental custody (e.g., Callow et al., 2017). This assumption has, however, been challenged by research indicating substantial variation in caregiving capacity (e.g., Willems et al., 2007) and child development (e.g., Collings & Llewellyn, 2012; Hindmarsh et al., 2015) among the pertinent families. Moreover, research has demonstrated high levels of contextual risk factors for caregiving and child developmental problems in the lives of families headed by parents with ID (Feldman & Aunos, 2020). For instance, parents with ID are often socioeconomically disadvantaged (Powell et al., 2017), and struggle with mental health problems (O’Keeffe & O’Hara, 2008), social isolation (Llewellyn & Hindmarsh, 2015), and parenting stress (Meppelder et al., 2015). Such risk factors are strongly linked to caregiving difficulties and negative child outcomes in the general population (e.g., Neppl et al., 2016; Plass-Christl et al., 2017; Stith et al., 2009). Indeed, many of the aforementioned risks associated with families of parents with ID have been found partly attributable to such contextual factors (Schuengel et al., 2017).

Another contextual factor that may be highly relevant for understanding caregiving and child developmental risks in families headed by parents with ID, is parental experiences of interpersonal trauma (IPT) in childhood. Such experiences include physical, sexual, and emotional abuse, neglect, and witnessing physical abuse in the family (e.g., Carlson et al., 2020; Wolfe et al., 2003). Additionally, prolonged, involuntary separations from caregivers have been emphasized as a form of IPT, especially for young children, based on empirical associations between such separations and adverse developmental outcomes (e.g., Jones-Mason et al., 2021). Meta-analytic findings among parents without ID have shown that IPT in childhood constitutes an important risk factor for negative caregiving behaviors, such as coercive and abusive parenting, inconsistent discipline, and physical punishment (Lotto et al., 2021; Van IJzendoorn et al., 2020). Childhood IPT has also been linked to adult mental health problems (e.g., Gardner et al., 2019), parenting stress (e.g., Lange et al., 2019; Lotto et al., 2021), and behavioral, emotional, and cognitive problems in one’s own children (Schickedanz et al., 2018). Beyond childhood, findings have linked IPT in adulthood to caregiving difficulties and parental aggression, and to child behavioral and cognitive problems (e.g., Chiesa et al., 2018; Vu et al., 2016). In sum, among parents *without* ID, parental IPT has been linked to the very same problems that are overrepresented among parents with ID and their children.

Notably, research also indicates that parents with ID are at especially high risk for IPT. The extent of elevated risk is, however, less clear. While some studies suggest extremely high levels of IPT (e.g., McGaw et al., 2007), other studies have reported relatively lower levels (e.g., Hellfritz, 2018). Additionally, some studies on parents with ID (e.g., Granqvist et al., 2014; Lindberg et al., 2017) have reported links between parental IPT and caregiving-relevant and child developmental outcomes, mirroring the links found among parents without ID. However, other studies have failed to establish corresponding links (e.g., Tymchuk & Andron, 1990). Despite these inconsistencies, no systematic review has offered synthesized knowledge regarding the prevalence of IPT among parents with ID, or its potential links to caregiving and child development outcomes.

Such knowledge would be valuable for several reasons. First, because studies on parents with ID often have small samples, the findings are hard to generalize. Second, professionals often struggle with assessing and treating caregiving risks among parents with ID. More thorough knowledge about the prevalence of IPT, and potential links to caregiving-relevant outcomes (e.g., caregiving behaviors, parental mental health), could inform this practice. Third, negative conceptions about the caregiving capacity of parents with ID are widespread (for a discussion, see e.g., Höglund et al., 2013), while the potential relevance of IPT for understanding their challenges is often overlooked (e.g., Mevissen et al., 2020). More solid knowledge about the role of IPT for caregiving-relevant and child outcomes in these families could contextualize caregiving difficulties and, thereby, counteract essentializing tendencies and discriminatory practices (Pacheco et al., 2021).

The overall aim of this systematic review was to provide the pertinent knowledge. More specifically, we set out to answer the following research questions: (a) How common are experiences of IPT among parents with ID, in general and in specific forms? Furthermore, are such experiences related to (b) caregiving-relevant outcomes and (c) developmental problems among their children?

## Method

A systematic review of extant literature was conducted in accordance with the updated Preferred Reporting Items for Systematic Reviews and Meta-Analyses statement (PRISMA 2020; Page et al., 2021). Article inclusion criteria were: peer-reviewed empirical articles in English, German, Swedish, or Norwegian; with abstract or alternate title in English; that report exposure to any form of IPT among parents with ID; based on systematic assessment of IPT. Eligible reports of IPT included: unspecified IPT (i.e., a global score, comprising any form of abuse or maltreatment); childhood physical, sexual, and emotional abuse, neglect, witnessing physical abuse in the family, and prolonged childhood separations from caregivers, including institutional rearing and out-of-home placement; or lifetime experiences of physical or sexual abuse. Studies pertaining to research question (a) were further required to be based on independent samples (i.e., studies were not allowed to share participants with other studies pertaining to the same research question). If publications reported on overlapping samples (i.e., the studies shared a proportion of their participants), we chose the largest sample as the independent one. If publications reported on the same sample (i.e., all participants were shared between the studies), the earliest publication was chosen. For research questions (b) and (c), sample overlap was allowed, given that the studies provided unique results pertaining to links between IPT and caregiving-relevant/child outcomes.

Exclusion criteria were: not reporting a clear operationalization of parental ID; not differentiating between ID and other disabilities; not differentiating between parents with ID and other adults with ID; not reporting IPT unambiguously (making it impossible to link IPT to the participants with ID); and not reporting original quantitative data (e.g., qualitative studies, case studies, reviews, conceptual pieces, or commentaries).

Electronic searches of MEDLINE, CINAHL (both EBSCOHost), PsycINFO, and PTSDpubs (both ProQuest) were completed on October 7 to 8, 2021, using the following terms: (mother OR maternal OR parent* OR caregiver) AND (“intellectual* disab*” OR “intellectual* limit*” OR “learning difficulties” OR “learning disability” OR “cognitive* impair*” OR “cognitive* limit*” OR “mental* retard*” OR “low IQ”) AND (maltreatment OR trauma OR abuse OR victimization OR neglect OR adverse OR ACE OR violence OR institutionalization). Terms were selected based on the authors’ knowledge of the literature on parents with ID, and were required to appear anywhere in the text. In the EBSCOHost databases, searches included related words and equivalent subjects. In the ProQuest databases, searches involved spelling and form variants of the search terms. All searches were limited to articles in peer-reviewed journals, published from 1970 to present. Relevant articles from the electronic search were subsequently entered into Google Scholar to identify papers citing these articles. This forward search also involved book chapters and dissertations. Additionally, a manual search of reference lists was conducted, as were a free hand search on Google. Forward and manual searches were completed on October 18, 2021.

**Figure 1.**
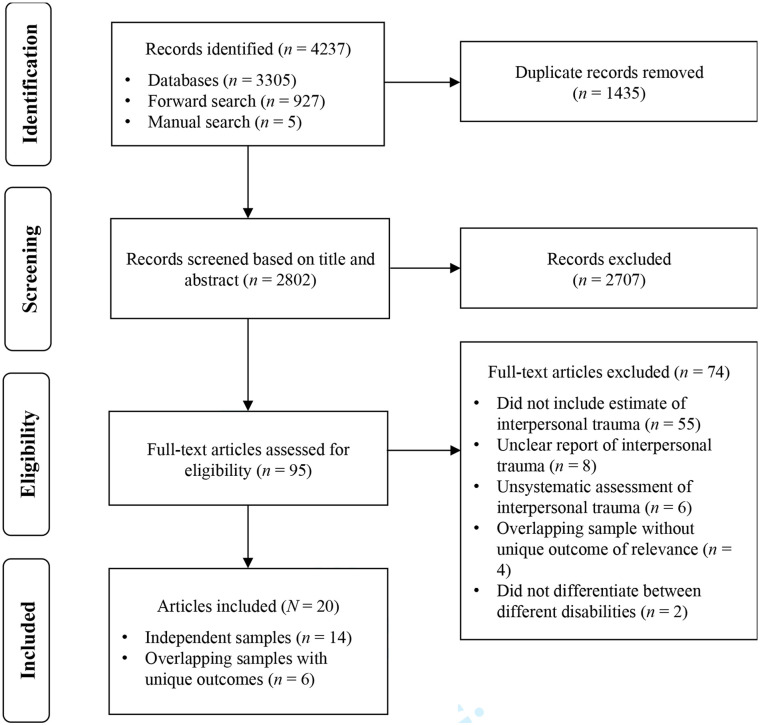
Study selection and review process. Flow chart based on PRISMA 2020, http://www.prisma-statement.org.

The search yielded 4,237 articles. Duplicates were removed with the support of Mendeley software, leaving 2,802 articles. Titles and abstracts were screened by MH for inclusion according to selection criteria, and remaining articles (*n* = 95) were assessed for eligibility by MH and TF, based on the full text. Disagreements were reviewed by PG, and discussed by all authors until consensus was reached. The most common reason for exclusion based on full text was lack of an estimate for parental IPT. Ultimately, 20 articles were included in the review (see PRISMA flow chart; Figure 1).

Data was extracted and summarized by MH using a standardized spreadsheet. Extracted data included: sample characteristics (e.g., sample type, number of participants with ID); study design, operationalization and severity of ID; timing and data source for IPT; form(s) and prevalence of IPT (unspecified, physical, sexual, and emotional abuse, neglect, witnessing physical abuse in the family, prolonged childhood separations from caregivers); comparison group without ID; caregiving-relevant outcomes; and child developmental outcomes. Study summaries were independently reviewed by PG and TF to establish accuracy. Ultimately, the quality of each included study was assessed by MH and TF, using the National Heart, Lung, and Blood Institute quality assessment tools for cohort and cross-sectional studies, and case-control studies (NIH, 2013). These instruments cover aspects of study quality relating to: formulation of research questions; representation of target population; sample size and study power; application of eligibility criteria across participants; participation and attrition rate; measurement of exposure and outcome; variation in levels of exposure; study timeframe; assessors’ knowledge about participants; and statistical analysis. The assessment yields an overall appraisal of each study (good/3, fair/2, or poor/1).

## Results

### Overview of the Studies

Table 1 summarizes key methodological facets of the included studies (*N* = 20), study findings pertaining to our research questions, and quality ratings of the studies. Information regarding these aspects, discussed below, can also be gleaned from a careful review of that table. A brief summary of critical findings can also be found in Table 2. The demographic characteristics presented are available in further detail in a separate Supplemental Appendix.

**Table 1.** Overview of Included Studies.

Study no.; Author (Year); Country; Language	Study Design	Sample Type; Number of Parents With ID	Operationalization and Severity of ID; Range, *M* (IQ), *SD*	Timing and Data Source for Parental IPT	Form(s) of IPT and Prevalence Reported	Comparison Group Without ID; Difference With Regard to IPT	Caregiving-Relevant Outcome (Measure) Investigated in Relation to IPT	Child Outcome (Measure) Investigated in Relation to IPT	Study Quality^a^
Independent Samples									
1; Gillberg and Geijer-Karlsson (1983); Sweden; English	RC	Convenience/ clinical; *n* = 15 mothers	Mothers had diagnosis of ID, IQ range = 55–70; *M*, *SD* not reported.	Childhood; Case records from social services.	PS: 34%	NA	NA	NA	2
2; Seagull and Scheurer (1986); United States; English	RC	Convenience/ clinical; *n* = 27 parents (74% mothers).	Parents had diagnosis of moderate ID—low borderline intellectual functioning. Tested IQ < 74; *M*, *SD* not reported.	Lifetime; notations about abuse experiences in clinic charts/CPS records.	U: 75%	NA	NA	NA	1
3; Tymchuk and Andron (1990); United States; English	C-C	Convenience/ clinical; *n* = 33 mothers.	Mothers had diagnosis of ID; *M* (neglecting/abusive mothers) = 69, *SD* = 4.5; *M* (non-neglecting/-abusive mothers) = 66, *SD* = 6.8.	Childhood; Dichotomous self-report question about general experiences of abuse/neglect. Answers were verified against agency records.	U: 51.5%	NA	Neglecting/abusive mothers had experienced descriptively less IPT than non-neglecting/-abusive mothers. Group difference not significant (χ^2^ = .77, *p* *=* .38)^a^	NA	2
4; Glaun and Brown (1999); Australia; English	RC	Convenience/ court; *n* = 12 mothers.	Mothers had diagnosis of ID; 11 mothers met criteria for mild ID, 1 for moderate ID; *M*, *SD* not reported.	Childhood; notations about abuse/neglect in clinical records/case files.	U: 75% SA: 58% N: 17%	NA	NA	NA	2
5; Ehlers-Flint (2002); United States; English	C-S	Convenience/clinical; *n* = 20 mothers.	Mothers had access to specialized services for parents with disabilities. Participant IQ was in the range 60–85; *M*, *SD* not reported.	Childhood; structured background interview including brief questions about abuse and neglect; open-ended questionnaire including questions about experiences of IPT.	U: 50% (based on brief questions). Prevalence of specific forms not reported.	NA	Parenting attitudes (PAQS). U was unassociated with PAQS score. PA/SA from open-ended questionnaire was linked to less enjoyment of child (*r*_pb_ = −.48, *p* < .05). EA was associated with difficulties disciplining child (*r*_pb_ = −.51, *p* < .05).	NA	1
Study no.; Author (Year); Country; Language	Study Design	Sample Type; Number of Parents With ID	Operationalization and Severity of ID; Range, *M* (IQ), *SD*	Timing and Data Source for Parental IPT	Form(s) of IPT and Prevalence Reported	Comparison Group Without ID; Difference With Regard to IPT	Caregiving-Relevant Outcome (Measure) Investigated in Relation to IPT	Child Outcome (Measure) Investigated in Relation to IPT	Study Quality^a^
6; Llewellyn et al. (2003); Australia; English	C-S	Convenience/clinical and court; *n* = 50 mothers.	Mothers had diagnosis of ID, a history of special education, or intellectual limitations noted by referring agency. IQ was in the range 42–97; *M* = 71.8, *SD* not reported.	Childhood; structured background interview including questions about experiences of PA/SA.	PA: 50%. SA: 46%.	NA	Physical and psychological health (SF-36). PA linked to more limitations in daily activities due to emotional problems (*t* = −2.29, *p* < .05)	NA	1
7; Elvish et al. (2006); United Kingdom; English	RC	Convenience/clinical; *n* = 82 parents (gender distribution not reported).	Parents had diagnosis of ID. Severity of ID and IQ statistics not reported.	Childhood; clinic charts.	SA: 37.5%	NA	Risk of caregiving difficulties (indicated by child removal). No link between SA and child removal.	NA	1
8; McGaw et al. (2010); United Kingdom; English	C-C	Convenience/clinical; *n* = 101 parents (96% mothers).	Parents had diagnosis of ID. Parents’ IQ was in the range 53–74; *M* = 67.6, *SD* = 5.5.	Childhood; case file information about IPT was verified against information from participants.	U: 51.5% PA: 30.7% SA: 23.8% EA: 41.6% PN: 12.9% EN: 11.9%	NA	Risk of caregiving difficulties (indicated by CPR registration or child removal). High-risk parents had experienced significantly more IPT (χ^2^ *=* 6.13, *p* *=* .01), especially EA (χ^2^ *=* 6.18, *p* *=* .01) and PA (χ^2^ *=* 10.19, *p* *=* .00).	NA	2
9; McConnell et al. (2011); Canada; English	RC	Convenience/court; n = 1170 caregivers (72.1% biological mothers).	Caregiver ID was considered present if case workers noted formal diagnosis, history of special education, or “observed” ID. No IQ data reported.	Childhood; dichotomous survey question about parent’s general experience of maltreatment/abuse, completed by caseworker.	U: 59.6%	Maltreatment-suspected caregivers without ID (*n* = 10,391). U: 20.3%.	Parental IPT increased risk of maltreatment substantiation (*OR* = 1.28, *p* < .001). Risk for maltreatment associated with caregiver ID decreased after control for psychosocial risk factors, including IPT (from *OR* = 1.51 to *OR* = 1.06), suggesting partial mediation by caregiver IPT.	NA	2
Study no.; Author (Year); Country; Language	Study Design	Sample Type; Number of Parents With ID	Operationalization and Severity of ID; Range, *M* (IQ), *SD*	Timing and Data Source for Parental IPT	Form(s) of IPT and Prevalence Reported	Comparison Group Without ID; Difference With Regard to IPT	Caregiving-Relevant Outcome (Measure) Investigated in Relation to IPT	Child Outcome (Measure) Investigated in Relation to IPT	Study Quality^a^
10; Emerson and Brigham (2013); United Kingdom; English	C-S	Community; *n* *=* 168 parents with ID that can be reliably tied to IPT data (gender distribution not reported).	Parental ID was considered present if health investigator noted current or previous need for special education. No IQ data reported.	Childhood; health care workers recorded information about experiences of abuse or out-of-home care from participants’ health care records. Notations were made in dichotomous form.	U: 42%	Single parents without ID (*n* = 5,088): U: 10%, group differences significant at *p* < .05.	Risk for parental mental illness associated with parental ID was decreased after control for exposure to adversities, including IPT (from *OR* = 1.50 to *OR* = 1.07), suggesting partial mediation by parental IPT.	NA	2
11; Granqvist et al. (2014); Sweden; English	C-C	Convenience/clinical; *n* = 23 mothers.	Mothers had diagnosis of mild ID and were entitled to specialist services for individuals with ID. *M*, *SD* for IQ not reported.	Lifetime; semi-structured 30-item interview covering a wide range of IPT. Responses were coded by two blinded researchers.	U: 91%; *M* (number of IPT events) = 2.88, *SD* = 1.95. PA: 61%^b^ SA: 58%^b^ EA: 38%^b^ N: 35%^b^ PS: 17%^b^	SES-matched mothers without ID (*n* = 25). U: 31%; *M* (number of IPT events) = 1.04, *SD* = 1.77. Group differences for U, PA, and SA significant at *p* < .01–.001, *d* = 0.77–1.00.	NA	Attachment security and disorganization (SAT). IPT among mothers with ID associated with lower attachment security (*r* *=* −.45, *p* < .05) and higher disorganization (*r* = .63, *p* < .01; *n* = 23).	2
Study no.; Author (Year); Country; Language	Study Design	Sample Type; Number of Parents With ID	Operationalization and Severity of ID; Range, *M* (IQ), *SD*	Timing and Data Source for Parental IPT	Form(s) of IPT and Prevalence Reported	Comparison Group Without ID; Difference With Regard to IPT	Caregiving-Relevant Outcome (Measure) Investigated in Relation to IPT	Child Outcome (Measure) Investigated in Relation to IPT	Study Quality^a^
12; Hellfritz (2018); Germany; German	C-S	Convenience/clinical; *n* = 127 parents with ID (97.6% mothers).	Parents had diagnosis of ID; IQ range = 43–73; *M* = 55.54, *SD* = 7.01.	Childhood; parents were asked dichotomous questions about general experiences of abuse and maltreatment, and of out-of-home placement, as a part of background interview.	U: 44.1% PS: 36.2%	NA	Maternal physical and psychological health (SF-12). IPT was associated with lower psychological health (*r* *=* −.20, *p* = .01). IPT was also associated with documented psychiatric diagnosis (*r* *=* .24, *p* < .01).	Motor (Bayley-III), cognitive (WPPSI-III/WISC-IV), and adaptive skills (ABAS-II). IPT unrelated to outcomes (*n* = 39–58 for different age categories).	2
13; Pacheco et al. (2021); Canada; English	RC	Convenience/court; n = 1,244 caregivers (91.3% female caregivers).	Caregiver ID was considered present if case workers noted formal diagnosis, history of special education, “observed” ID, or suspected ID. No IQ data reported.	Childhood; dichotomous survey question about parent’s experience of out-of-home care/institutional rearing, completed by caseworker.	PS: 35.5%	Maltreatment-suspected caregivers without ID (*n* = 14,736). PS: 10.3%. Group difference significant at *p* *<* .00	NA	NA	2
14; McConnell et al. (2021a); Canada; English	C-S	Convenience/clinical; *n* = 91 parents (90.1% mothers).	Parents had access to specialized services for people with ID. No IQ data reported.	Childhood; five questions from ACE questionnaire, four of which were IPT-specific, were asked in interview form.	U: 81%; *M* (number of IPT events) = 2.29, *SD* = 1.62. PA: 50.0% SA: 36.4% EA: 54.0% WAF: 48.3% (based on *n* = 85)	NA	Self-rated parenting role satisfaction (4 Likert-questions about enjoyment of parenthood), emotional warmth, and punitive discipline (PBDQ). Total IPT predictive of higher parenting role satisfaction (β = .29, *p* < .05), and unrelated to emotional warmth and punitive discipline. 40.7% did not complete punitive discipline scale.	NA	2
Study no.; Author (Year); Country; Language	Study Design	Sample Type; Number of Parents With ID	Operationalization and Severity of ID; Range, *M* (IQ), *SD*	Timing and Data Source for Parental IPT	Form(s) of IPT and Prevalence Reported	Comparison Group Without ID; Difference With Regard to IPT	Caregiving-Relevant Outcome (Measure) Investigated in Relation to IPT	Child Outcome (Measure) Investigated in Relation to IPT	Study Quality^a^
Overlapping Samples									
15; McConnell et al. (2003); Australia; English (sample overlap with no. 6).	C-S	Convenience/clinical and court; *n* = 40 mothers.	Mothers had a diagnosis of ID, or a history of special education, or intellectual limitations noted by referring agency. Screened IQ was in the range 42–97; *M* = 71.8, *SD* = 11.91.	Childhood; structured background interview including questions about general experiences of physical/sexual abuse.	PA: 60% SA: 57%	NA	NA	Child physical, self-help, social, academic, and communicative development (Developmental Profile II). IPT was unrelated to child developmental scores.	2
16; McGaw et al. (2007); United Kingdom; English (sample overlap with no. 8)	C-S	Convenience/Clinical; *n* = 49 parents (69.1% mothers). 77.6% were in a relationship with another participant.	Mothers had IQ in range 53–80 (*M* = 70.02, *SD* = 6.97). Fathers had IQ in range 62–90 (*M* = 77.1, *SD* = 8.72).	Childhood; questions from structured trauma questionnaire (CTQ) were asked in interview form.	U: 79.6% (mothers: 83.3%, fathers: 73.3%) PA: 44.9% SA: 40.8% EA: 57.1% PN: 30.6% EN: 51.0%	NA	Parental psychopathology (Mini PAS-ADD). Experiences of SA were associated with parental psychopathology (χ^2^ = 8.61, *p* < .00)*.	NA	2
17; Feldman et al. (2012); Canada; English (same sample as no. 9)	C-S	Convenience/court; n = 1,170 caregivers (72.1% biological mothers).	Caregiver ID was considered present if case workers noted formal diagnosis, history of special education, or “observed” ID. No IQ data reported.	Childhood; dichotomous survey question about parent’s general experience of maltreatment/abuse, completed by caseworker	U: 59.6%	NA	NA	IPT predicted child learning/developmental problems (β = .58, *SE* = .15, *OR* = 1.78, *p* < .001). Data based on case worker notations.	2
18; Lindberg et al. (2017); Sweden; English (same sample as no. 11)	C-C	Convenience/Clinical; *n* = 23 mothers.	Mothers had diagnosis of mild ID and were entitled to specialist services for individuals with ID. *M*, *SD* for IQ not reported.	Lifetime; semi-structured 30-item interview covering a wide range of IPT. Responses were coded by two blinded researchers.	U: 91%; *M* (number of IPT events) = 2.88, *SD* = 1.95.	SES-matched mothers without ID (*n* = 25). U: 31%; *M* (number of IPT events) = 1.04, *SD* = 1.77. Group difference significant at *p* < .001, *d* = 1.00.	Maternal sensitivity (ASS). Total IPT among specifically mothers with ID was associated with lower sensitivity (*r* = −.43, *p* < .05).	NA	2
Study no.; Author (Year); Country; Language	Study Design	Sample Type; Number of Parents With ID	Operationalization and Severity of ID; Range, *M* (IQ), *SD*	Timing and Data Source for Parental IPT	Form(s) of IPT and Prevalence Reported	Comparison Group Without ID; Difference With Regard to IPT	Caregiving-Relevant Outcome (Measure) Investigated in Relation to IPT	Child Outcome (Measure) Investigated in Relation to IPT	Study Quality^a^
19; McConnell et al. (2021b); Canada; English (sample overlap with no. 13)	RC	Convenience/court; n = 1,000 caregivers (87.6% biological mothers).	Caregiver ID was considered present if case workers noted formal diagnosis, history of special education, “observed” ID, or suspected ID. No IQ data reported.	Childhood; dichotomous survey question about parent’s experience of out-of-home care/institutional rearing, completed by caseworker.	PS: 32.4%	Maltreatment-suspected caregivers without ID (*n* = 14,980): PS: 8.9%. Group difference significant at *p* < .00.	Risk for maltreatment associated with caregiver ID decreased after control for psychosocial risk factors, including IPT (from *OR* = 1.80 to *OR* = 1.31), suggesting partial mediation by caregiver IPT.	NA	2
20; Hammarlund et al. (2021); Sweden; English (same sample as no. 11)	C-C	Convenience/clinical; *n* = 23 mothers.	Mothers had diagnosis of mild ID and were entitled to specialist services for individuals with ID. *M*, *SD* for IQ not reported.	Lifetime; semi-structured 30-item interview covering a wide range of IPT. Responses were coded by two blinded researchers.	U: 91%; *M* (number of IPT events) = 2.88, *SD* = 1.95.	SES-matched mothers without ID (*n* = 25). U: 31%; *M* (number of IPT events) = 1.04, *SD* = 1.77. Group difference significant at *p* < .001, *d* = 1.00.	Interpretation of infant facial emotions (IFEEL). Total IPT among mothers with ID associated with misinterpretations of shame (*r*_pb_ = .47, *p* < .05), which was linked to lower child attachment security (*r*_pb_ = .41, *p* < .05) and higher disorganization (*r*_pb_ = .43, *p* < .05)	NA	2

*Note.* ACE = adverse childhood experiences; ASS = Ainsworth sensitivity scale; C-C = case-control; CPR = child protection register; CPS = child protection services; C-S = cross-sectional; CTQ = childhood trauma questionnaire; EA = emotional abuse; EN = emotional neglect; ID = intellectual disability; IPT = interpersonal trauma; IQ = intelligence quotient; IFEEL = infant facial expressions of emotion from looking at pictures test; Mini PAS-ADD = psychiatric assessment schedule for adults with a developmental disability, short-form; NA = not applicable/available; OR = odds ratio; PA = physical abuse; PAQS = parenting attitudes Q-sort; PBDQ = parenting behaviors and dimensions questionnaire; PN = physical neglect; PS = prolonged childhood separations from caregivers; SA = sexual abuse; SAT = separation anxiety test; SES = socio-economic status; SF-12/36 = MOS 12-/36 item short form health survey; U = unspecified IPT; WAF = witnessing abuse in the family.

a Article does not provide a specific chi-square statistic, but reports the information necessary for calculation. The displayed statistic has been computed by review authors.

b Results for specific forms are not reported in the original article, but review authors have access to raw data.

* Study quality: 1 = Poor; 2 = Fair.

**Table 2.** Summary of Critical Findings.

Study Characteristics	Prevalence	Caregiving-Relevant and Child Developmental Outcomes
Studies displayed notable variation in sample type and size, operationalization of ID, and assessment of IPT.	Findings indicate that a majority of parents with ID have experienced some form of IPT.	A majority of the pertinent studies found links between IPT and adverse caregiving-relevant outcomes. Links to parental mental health problems were particularly consistent.
Only a small minority of the studies employed thorough assessments of IPT and/or outcomes.	Unspecified IPT was very common, even compared to other groups at elevated risk.	Findings pertaining to child developmental outcomes were scant and inconsistent.
There is limited data on several specific forms of IPT, and on cumulative and lifetime IPT, among parents with ID.	Findings for specific forms of IPT also indicate high prevalence. Experiences of abuse and prolonged childhood separations from caregivers were especially common.	

*Note.* ID = intellectual disabilities; IPT = interpersonal trauma.

The included studies were published between 1983 and 2021, with a majority (*n* = 12) from 2010 and onwards. All studies originated in Western high-income countries, including Canada (*n* = 5), United Kingdom (*n* = 4), Sweden (*n* = 4), Australia (*n* = 3), the United States (*n* = 3), and Germany (*n* = 1). Three study designs were detected, with cross-sectional design the most common (*n* = 8), followed by retrospective cohort (*n* = 7) and case-control (*n* = 5). All studies were published in English, except for one, which was published in German.

Fourteen studies were based on independent samples, while an additional six studies involved overlapping or identical samples with previously unreported investigations of links between IPT and caregiving-relevant or child developmental outcomes. Most studies (*n* = 14; 10 independent studies) employed convenience samples from clinical settings, such as specialized support centers for parents with ID, while five studies (three independent) employed samples from court or child protection settings. One sample was community-based. The number of participants with ID in the studies varied substantially; from 12 participants in the smallest study, to 1,244 participants in the largest one. Seven studies (four independent) involved comparison groups, comprising between 25 and 14,980 participants.

Concerning operationalization of ID, 10 studies (eight independent) explicitly included only parents with a formal diagnosis of ID. The vast majority of parents in these studies had mild ID (IQ 50–69), although one study (Elvish et al., 2006) did not report severity of ID, and five studies also included a small minority of parents with either borderline intellectual functioning (IQ 70–85; one study), moderate ID (IQ 35–50; one study), or a small minority from both these categories (three studies). An additional seven studies (four independent) employed a wider operationalization and included parents who either had a formal diagnosis of ID, a history of special education, or ID as observed or suspected by caseworkers. The remaining three studies (two independent) based eligibility on participants’ access to specialized support services for parents with ID. Descriptive statistics from studies employing the latter two operationalizations indicated substantial variation with regard to ID severity, and a relatively high proportion of parents with borderline intellectual functioning.

Regarding timing of IPT, 16 studies (12 independent) assessed childhood prevalence, while 4 studies (two independent) assessed lifetime prevalence. There was notable diversity in how the assessments were conducted. Nine studies (seven independent) used clinic chart information, or case files from social/child protection services. Five studies (two independent) used structured or semi-structured instruments, or parts of such instruments, as a basis for interviews, and three studies (two independent) used brief screening questions in a structured background interview. Two studies (independent) verified information from participants against case files, and one study (independent) used two different instruments.

The forms of trauma studied also varied substantially. Five of the independent studies only reported estimates for unspecified IPT, while five studies included both specific forms and an unspecified estimate comprising any form of IPT. An additional four independent studies exclusively reported results for a few specific forms. The most common specific forms of trauma studied were sexual abuse (*n* = 6), physical abuse (*n* = 4), and prolonged separations from caregivers (*n* = 4). Reports of emotional abuse and neglect were found in three studies. One study provided data for witnessing physical abuse in the family.

Thirteen studies examined links between IPT and caregiving-relevant outcomes. Five of these studies used child removal, child protection involvement, or child maltreatment substantiation as proxy variables for caregiving difficulties. Another four studies reported outcomes related to caregivers’ mental health. Yet another two studies reported self-rated parenting attitudes and/or parental behavior, and an additional two studies examined caregiving-relevant outcomes through observational methods. Four studies examined links between IPT and child developmental outcomes. Three of these studies reported such outcomes based on standardized assessment procedures, and one study investigated such outcomes based on case file data.

With regard to demographic characteristics, 16 studies provided data on gender. The proportion of female participants in these studies ranged from 69% to 100%, with 14 studies involving ≥90% females. Two additional studies mainly included females but did not report on exact gender distribution, and two studies provided no gender data at all. Participant age was reported in only 12 studies, and ranged from 16 to 55 years, with a typical mean age in the range 30 to 35 years. Reporting of participant ethnicity was unsatisfactory, with only nine studies providing such data. While these reports overall indicated a majority of White participants, they were generally too undetailed to offer a nuanced picture. For instance, six studies only reported the proportion of “White/Not White” or “Aboriginal/Not Aboriginal.”

Reports were similarly scarce with regard to co-occurring conditions among the participants. Only four studies explicitly examined co-occurring physical or sensory disabilities, and found rates of 2% to 16%. Additionally, nine studies examined co-occurring psychiatric conditions or mental health issues, and found such conditions to be present among 18% to 71% of participants, with seven studies reporting rates of ≥40%.

A clearer picture appeared with regard to the parents’ living conditions. For instance, 18 studies presented data pertaining to socio-economic status, and consistently indicated that a vast majority of the participants lived under harsh socio-economic conditions (e.g., low income, unemployed, dependent on welfare, lived in public housing). Relatedly, studies reporting on participants’ social situation typically found loneliness and isolation to be common. For instance, 12 of the 14 studies that provided data on partner status found that ≥40% of participants lived without a partner.

### Methodological Quality of the Studies

Sixteen of the studies were rated as “fair” and four as “poor,” using the NIH quality assessment tools. The most common methodological limitations were: lack of blinded outcome assessors (*n* = 11); only one single measurement of key exposure variables (*n* = 10); lack of sample size justifications and/or effect size estimates (*n* = 9); measuring exposure and outcome on the same occasion (*n* = 9); and not controlling for confounds (*n* = 8; detailed assessments are available in a separate Supplemental Appendix).

### Main Findings of the Studies

Mirroring our research questions, the main findings are organized into prevalence of unspecified IPT, prevalence of specific forms of IPT, associations with caregiving-relevant outcomes, and associations with child developmental outcomes. We restrict the presentation to a narrative summary because of the notable heterogeneity regarding sample type, operationalization of parental ID, assessment of IPT, and reporting of IPT data. The overall proportion of parents subjected to unspecified IPT from clinical samples could, however, be calculated. This was because there were enough studies for such an estimate to be meaningful, and because the pertinent studies reported either the sheer number of parents subjected to such experiences, or a specific proportion of the total number of parents (enabling calculation of sheer numbers). If calculation of sheer numbers yielded uneven numbers, indicating that the reported proportions had been rounded up or down, we consistently rounded down to ascertain conservative estimates. The overall proportion was then calculated as the total number of trauma-subjected parents, divided by the total number of participants within clinical samples. In addition to this estimate, we provide the weighted median proportion for unspecified IPT in clinical samples and across all independent samples, as well as for specific forms for which at least four unambiguous estimates were available.

#### Prevalence of unspecified IPT

Ten studies provided estimates for *unspecified IPT*. These studies came from seven clinical samples, two court-based samples, and one community-based sample, and mainly regarded childhood experiences (eight studies). The levels of IPT were consistently very high, with all but two studies reporting such experiences among at least 50% of the parents with ID, and half of the studies reporting prevalence rates of ≥60% (Glaun & Brown, 1999; Granqvist et al., 2014; McConnell et al., 2011, 2021a; Seagull & Scheurer, 1986; *n* = 12–1,170). For clinical samples, the overall proportion of parents with ID subjected to unspecified IPT was 58% (combined *n* = 416), and the weighted median proportion was 51.5%. The highest clinical prevalence estimates (Granqvist et al., 2014; McConnell et al., 2021a) were based on comparatively comprehensive assessments of IPT, whereas the lowest estimate (Hellfritz, 2018) was based on a relatively simple assessment. Across all independent samples, the weighted median proportion of parents with ID subjected to unspecified IPT was 59.6%.

Three of the studies reporting unspecified IPT also involved comparison groups, which were reasonably matched for socioeconomic status. Unspecified IPT was markedly more common among parents with ID in all three studies. In a large court sample, McConnell et al. (2011) found a childhood prevalence of about 60% for parents with ID and 20% for parents without ID. In the community-based sample, childhood prevalence rates were 42% for single parents with ID and 10% for single parents without ID (Emerson & Brigham, 2013). In a clinical sample, Granqvist et al. (2014) found lifetime prevalence rates of 91% for mothers with ID, and 31% for comparison mothers. Although the proportion of IPT-subjected parents varied across sampling contexts, parents with ID were consistently found to be at heightened risk for IPT.

#### Prevalence of specific forms of IPT

With regard to *physical abuse*, four studies using clinical samples reported prevalence rates of 31% to 61%. The highest estimate came from the small-sample study by Granqvist et al. (2014), focusing on lifetime prevalence. Relatively lower estimates were reported from the larger studies, focusing on childhood experiences (Llewellyn et al., 2003; McConnell et al., 2021a; McGaw et al., 2010). The weighted median proportion was 50%.

Regarding *sexual abuse*, five of the six estimates pertained to childhood experiences, and the reported prevalence levels ranged between 24% and 58%. The highest proportions were found in small-sample studies, from court and clinical contexts (Glaun & Brown, 1999; Granqvist et al., 2014). Comparatively lower proportions of between a third and just less than half were reported in larger studies based on clinical samples (Elvish et al., 2006; Llewellyn et al., 2003; McConnell et al., 2021a). The lowest proportion reported was about 24%, also from a clinical sample (McGaw et al., 2010). The weighted median proportion was 37.5%.

Studies examining *prolonged childhood separations from caregivers* reported prevalence rates of 17% to 36%. The highest estimate was reported in the large clinical-sample study by Hellfritz (2018), which employed an operationalization that included both prolonged out-of-home placements and repeated moves between caregivers. Pacheco et al. (2021) reported an almost identical estimate in their large court-based study, with just above 35% of parents with ID having childhood experiences of out-of-home placement, compared to 10% of the parents without ID. Gillberg and Geijer-Karlsson (1983) also reported a very similar estimate in a small clinical sample, with 34% of the mothers with ID having been partly or wholly raised in institutions. The lowest proportion (17%) came from Granqvist et al.’s (2014) small, clinical sample of mothers. This study did, however, employ a somewhat stricter definition of IPT-relevant separations, including only repeated separations and/or loss of caregiver through death. The weighted median proportion was 35.5%.

Estimates for childhood *emotional abuse* came exclusively from clinical samples, and ranged from 38% to 54%. The higher estimates came from two relatively large studies (McConnell et al., 2021a; McGaw et al., 2010), while the lowest estimate came from the small-sample study by Granqvist et al. (2014).

The highest estimate of *neglect* (35%) was reported in the small clinical-sample study by Granqvist et al. (2014). A notably lower prevalence (17%) was reported in the very small court-sample study by Glaun and Brown (1999). This study did, however, employ an unusually strict definition of neglect in terms of particularly marked experiences of deprivation. The lowest estimates of neglect came from the larger clinical-sample study by McGaw et al. (2010), in which 12% and 13% of the parents were found to have experiences of emotional and physical neglect, respectively. Unfortunately, the degree of overlap between parents subjected to these forms of neglect was not reported.

One independent study provided an unambiguous estimate for *witnessing physical abuse in the family* (McConnell et al., 2021a). In this comparatively large clinical-sample study, 48% of the parents reported exposure to such events during childhood.

#### Parental IPT and caregiving-relevant outcomes

There were inconsistent associations between reports of parental IPT and child removal, child protection involvement, and child maltreatment. For instance, Tymchuk and Andron (1990) found that unspecified childhood IPT was descriptively *less* common among clinically enrolled abusive and/or neglecting mothers with ID than among non-neglecting/-abusive mothers with ID. This study did, however, use a comparatively simple assessment of IPT. Examination by review authors also showed the group difference to be statistically insignificant (Yate’s-corrected χ^2^ = .77, *p* = .38). Similarly, Elvish et al. (2006), also using a clinical sample, found no difference with regard to IPT among parents with ID who subsequently did or did not have their children removed. Notably, this study only assessed parental experiences of childhood sexual abuse. The larger study by McGaw et al. (2010), employing a more comprehensive assessment of IPT, found contrasting results; parents with childhood experiences of IPT were at significantly higher risk for child removal, or registration of a child on the child protection register. The associations were particularly strong for parents who reported experiences of emotional and physical abuse. Relatedly, the very large court-based study by McConnell et al. (2011) found that parental experiences of unspecified childhood IPT contributed to the elevated risk for child maltreatment substantiation among parents with ID. Notably, this risk was also markedly reduced, and rendered statistically insignificant, following control for a combination of contextual risk factors, including parental IPT. A similar result was found in another large court-based study (McConnell et al., 2021b), in which control for a combination of contextual risk factors, including parental experiences of prolonged childhood separations from caregivers, reduced the elevated risk for child maltreatment substantiation associated with parents with ID. In this study, however, parental ID remained a statistically significant predictor of child maltreatment substantiation.

Regarding parents’ mental health, all pertinent studies indicated links between parental IPT and negative mental health outcomes. For instance, Llewellyn et al. (2003) found that mothers with experiences of childhood physical abuse self-reported more limitations in daily activities due to emotional problems. Similarly, Hellfritz (2018) found associations between unspecified childhood IPT and lower self-reported psychological health and a higher risk of psychiatric diagnosis. Further, McGaw et al. (2007) found that parental experiences of childhood sexual abuse were associated with parental psychopathology. Finally, Emerson and Brigham (2013) found, in the community-based study, that the observed heightened risk for mental illness among parents with ID was eliminated after control for exposure to adversities, including unspecified childhood IPT and exposure to domestic violence. Due to data limitations, it was however not possible to determine whether the parents were direct victims or witnesses to the domestic violence.

With regard to self-reported parenting attitudes or behavior, the small clinical-sample study by Ehlers-Flint (2002) found moderate to strong associations between childhood physical/sexual abuse and less self-reported enjoyment of one’s own child, and between emotional abuse and difficulties in disciplining one’s own child. Unfortunately, the proportion of parents subjected to these forms of abuse was not reported. This study also employed two measures for IPT, of which one (for unspecified childhood IPT) was unrelated to parenting attitudes. Contrasting findings were reported in the clinical-sample study by McConnell et al. (2021a), in which cumulative childhood IPT was *positively* related to parenting role satisfaction, and unrelated to parental emotional warmth and punitive discipline. While this study was considerably larger and used a fairly stringent measure of IPT, almost half of the participants, including almost all parents who did not have full custody of their child, did not complete the punitive discipline scale.

Lastly, two clinical-sample studies examined IPT in relation to observational measures. In the first, Lindberg et al. (2017) found a moderate to strong negative association between cumulative lifetime IPT among parents with ID, and sensitivity to their children’s signals. Lower parental sensitivity was in turn trend-significantly related to higher levels of child attachment disorganization. In the second, Hammarlund et al. (2021) found a moderate to strong positive association between cumulative lifetime IPT among parents with ID, and a proclivity to misinterpret shame from infant facial expressions. This proclivity was, in turn, associated with lower levels of child attachment security and higher levels of attachment disorganization. While these studies used comprehensive assessments of IPT as well as of caregiving outcomes, both were based on the same small sample.

#### Parental IPT and child developmental outcomes

Associations between parental IPT and child developmental outcomes were inconsistent. A court-based study (Feldman et al., 2012) found that parental experiences of unspecified childhood IPT were strongly predictive of child learning and developmental problems. While this study was among the largest reviewed, the pertinent regression model displayed poor fit with data, and the study used fairly simple assessments of both IPT and outcomes. Relatedly, Granqvist et al. (2014) found that parents’ cumulative lifetime IPT score was moderately to strongly associated with lower child attachment security, and strongly associated with child attachment disorganization. This study used rigorous assessments of both IPT and outcomes, but the sample was small. The large clinical-sample study by Hellfritz (2018), on the other hand, focusing on child motor, cognitive, and adaptive skills, found no associations between parental childhood IPT and negative child developmental outcomes. Similarly, McConnell et al. (2003) found no relationship between parental experiences of childhood physical or sexual abuse, and children’s status across five developmental domains. While these studies both used validated outcome measures, they employed simple assessments of IPT.

## Discussion

The aim of this review was to fill a critical gap in the literature by synthesizing data on (a) the prevalence of experiences of IPT among parents with ID, and on links between such experiences and (b) caregiving-relevant and (c) child developmental outcomes. With regard to research question (a), the review clearly indicates that a majority (>50%) of parents with ID have experienced some form of IPT, although exact prevalence is uncertain and may vary somewhat across contexts (clinical, court, or community). Experiences of abuse and prolonged childhood separations from caregivers were especially common. Regarding question (b), findings were mixed, but most of the pertinent studies indicated links between IPT experiences and adverse caregiving-related outcomes among parents with ID. Concerning question (c), links to child developmental outcomes were more ambiguous. These main findings are discussed below.

### Prevalence of IPT Among Parents with ID

Overall, the review found IPT to be remarkably common among parents with ID. In fact, even the lowest reported estimate of unspecified IPT (42%; the community-based study by Emerson and Brigham, 2013) is higher than corresponding estimates for other populations at heightened risk for IPT, such as patients with severe psychopathology (cf. Gatov et al., 2020; 32%). Furthermore, although our combined prevalence estimates (59.6% across all reviewed studies; 51.5%–58% across clinical studies) should be interpreted with caution, given the heterogeneity of assessments and observed prevalence rates, it seems unlikely that the estimates are inflated. Indeed, they may actually constitute underestimations of the true prevalence, because most included studies employed relatively simple assessments of IPT, implying a heightened risk for under-detection (Glaesmer, 2016). Conversely, the studies using more comprehensive assessments of IPT typically reported higher prevalence rates.

Generally, the review also indicated that most specific forms of IPT are overrepresented among parents with ID. For instance, the weighted median proportions for *physical abuse* (50%) and *sexual abuse* (37.5%) are notably higher than comparable population norms (cf. Moody et al., 2018; Stoltenborgh et al., 2015). Similarly, the estimates for *emotional abuse* (38%–54%) and *witnessing physical abuse in the family* (48%) both exceed population estimates from comparable countries (cf. Giano et al., 2020; Stoltenborgh et al., 2015), although the limited data for these specific forms render generalizations less certain.

An exceptionally high proportion of parents with ID was also found to have experiences of *prolonged childhood separations from caregivers*. To illustrate, the weighted median proportion of 35.5% is 4 to 10 times higher than comparable proportions of individuals with childhood experiences of out-of-home care in the pertinent countries (cf. e.g., Berlin, 2020; O’Donnell et al., 2016). Most of these separations were likely initiated to protect the individuals from other adverse experiences in childhood (e.g., severe abuse or neglect), and may thus have been beneficial relative to risks in their previous caregiving context. Regardless, the findings clearly show that the childhood histories of many parents with ID are characterized by caregiving instability. This is an important finding, since such instability has in itself been linked to notable developmental risks (see e.g., Jones-Mason et al., 2021).

The only specific form of IPT for which prevalence levels were not particularly elevated among parents with ID was childhood *neglect* (12%–35%; cf. Moody, 2018). This is surprising, since many individuals with ID are raised in environments involving a heightened risk for neglect (Slack et al., 2004). Neglect is, however, comparatively difficult to assess (Allnock, 2016), and the pertinent studies used different definitions and assessments of the construct. Additionally, two of the studies were among the smallest in the selection, so conclusions regarding the prevalence of neglect should be drawn tentatively.

### Associations With Caregiving-Relevant Outcomes

Overall, the reviewed studies indicated links between IPT and adverse caregiving-relevant outcomes. Yet, findings were sprawling and a coherent picture was obstructed by marked methodological discrepancies. The most consistent evidence regarded parental mental health, with all pertinent studies linking IPT among parents with ID to mental health problems. This finding is important, as such problems heighten the risk for negative parenting practices (e.g., Kohl et al., 2011), and may interfere with parents’ capacity for collaborating with support services. Notably, the study by McConnell et al. (2011) also found that parental mental health problems partly mediated maltreatment investigation outcomes for parents with ID, including maltreatment substantiation, case disposition, and application to the child welfare court.

However, only two studies (McConnell et al., 2011; McGaw et al., 2010) provided direct evidence of IPT among parents with ID as a risk factor for child protection involvement, child removal or child maltreatment substantiation, while one additional study provided indirect evidence (McConnell et al., 2021b). Taken together, these studies indicate a link between IPT among parents with ID and child-protection involvement/child maltreatment. Crucially, these three studies also displayed important methodological strengths compared to studies that did not find a corresponding link (e.g., larger samples, more extensive mapping of IPT). Yet, conclusions should be drawn with caution; not least since the use of combined psychosocial-risk scores in one of the studies prevents closer examination of specific contributions of parental IPT per se.

Findings with regard to self-reported parenting attitudes or behaviors were inconsistent, and hard to evaluate due to the scarcity of data and study limitations (e.g., very small sample size; high attrition rates for questions about negative parenting behaviors). The reliance on self-reports of parental behaviors is also a notable limitation, because the correspondence between such reports and actual parenting behavior is particularly modest among parents who, like many parents with ID, have low socioeconomic status or struggle with mental health problems (Herbers, 2017). In contrast, the two reports (from the same project) based on observational measures found theoretically expectable associations between cumulative parental IPT and negative caregiving-related outcomes (lower sensitivity, shame misinterpretations; Hammarlund et al., 2021; Lindberg et al., 2017). Here, generalizations are instead hampered by the small sample size.

### Associations With Child Developmental Outcomes

Findings provided weak evidence for links between parental IPT and child developmental outcomes, and interpretation is again complicated by study limitations. It should, however, be noted that the only study employing comprehensive assessments of both parental IPT and child outcomes did report an association between the two (Granqvist et al., 2014). Yet, no firm conclusions can be drawn on the basis of this study alone. Additionally, while methodological disparities may be one reason for inconsistent findings, child development is determined by multiple factors. For instance, children of parents with ID are also at heightened risk for developmental delays due to genetic predispositions (e.g., Vissers et al., 2016). Thus, difficulties observed among the children in the pertinent studies may have had different etiological roots, making them harder to link to specific predictors.

### Methodological Considerations and Future Research Needs

With regard to study quality, none of the studies received a rating better than “fair.” While this may indicate suboptimal study quality by objective scientific standards, it should be borne in mind that recruitment is a ubiquitous challenge in research on parents with ID. Consequently, researchers are forced into a trade-off between better assessments (yielding small samples) and larger samples (necessitating simpler assessments). Partly for these reasons, a recent systematic review of parent training interventions for parents with ID rated most evidence as being of low quality (Coren et al., 2018). Such conclusions should not be viewed as negative judgments about the research community, but as reflections of the many difficulties inherent to conducting research in this area.

Beyond global quality ratings, the reviewed studies displayed a number of specific limitations. Since these limitations are closely tied to future research needs, they are discussed together in the following. A summary of future research needs is also presented in Table 3, together with the implications of our findings for policy and practice. First, only a minority (23%) of the pertinent studies examined a broad range of specific forms of IPT in relation to caregiving-relevant outcomes. This is unfortunate, since research indicates that specific forms of IPT may be differentially related to caregiving (Buisman et al., 2019). Future research should address this limitation by employing more comprehensive assessments of IPT, including its multiple forms.

**Table 3.** Implications for Research, Policy, and Practice.

Research	Policy and Practice
Future studies should employ more comprehensive assessments of IPT, including specific forms of IPT, cumulative IPT, and IPT occurring after childhood.	Practitioners should be aware that IPT-experiences are very common among parents with ID, and support services should screen parents for such experiences.
Future studies should employ observational measures of caregiving, and longitudinal designs.	Practitioners should be open to viewing parents’ difficulties in the light of past and current hardships, rather than as mere consequences of their ID.
There is a need for empirical research that involves fathers with ID, and parents with ID from non-clinical and non-Western populations. There is also a need for data regarding participant ethnicity and co-occurring physical/sensory disabilities.	Practitioners should be offered training in trauma-informed care, and traumatized parents with ID should be offered suitable therapeutic interventions.
Researchers should strive to supplement parental self-reports with objective data on IPT during the first years of life.	

*Note*. ID = intellectual disabilities; IPT = interpersonal trauma.

Second, a similarly small minority (25%) of the pertinent studies examined the role of cumulative parental IPT for caregiving-relevant or child outcomes. This is a crucial limitation, because research on childhood adversities has repeatedly demonstrated a dose-response relationship between the number of adversities experienced and negative caregiving-relevant outcomes (e.g., Lange et al., 2019). Additionally, an even smaller minority (19%) of the pertinent studies examined the role of lifetime (rather than childhood) IPT. This is also noteworthy, because IPT occurring after childhood (e.g., being victim to intimate partner violence) has also been found to affect caregiving and child outcomes (e.g., Chiesa et al., 2018; Vu et al., 2016). Consequently, we strongly encourage future research to include assessments of cumulative IPT, and IPT occurring after childhood.

Third, only a small minority of the studies (15%) used observational methods to tap caregiving-relevant outcomes. This is also a notable limitation, because such methods are usually considered superior to other methods for assessing caregiving (e.g., Hawes & Dadds, 2006). Thus, we urge future research to use observational methods.

Fourth, estimates of specific forms of IPT were largely based on clinical samples, and generalizability to parents with ID in other contexts is thus uncertain. This is particularly important regarding parents with ID in the larger community, as community-based estimates for specific forms of IPT were completely absent. Additionally, the studies were conducted in Western high-income countries and primarily involved mothers. This illustrates a dramatic research gap regarding IPT among parents with ID in the larger community, in non-Western countries, and among fathers. Future research should strive to fill these gaps.

Fifth, there was a lack of data on participant ethnicity, and the generalizability of the findings to specific ethnical contexts thus remains uncertain. Similarly, very few studies examined the potential role of co-occurring physical or sensory disabilities among the parents. Future research should aim to clarify these issues through more thorough mappings of such constructs.

Sixth, most studies relied on self-reports, or chart information based on such reports. While this is more or less customary in research on IPT, it implies that potential experiences of IPT during the earliest years are likely to go undetected. This is unfortunate, since IPT during the first years of life is associated with hampered neurocognitive development, even after control for contextual variables (e.g., Enlow et al., 2012). Crucially, this also hints at the multidetermined etiology of ID, and the possibility of severe early IPT as an etiological factor behind ID among some parents, rather than a coexisting risk factor for subsequent outcomes. Although methodologically difficult to tackle, future studies should strive to address this issue.

Lastly, none of the included studies employed longitudinal designs. This is also an important limitation, as such designs would enable a more nuanced examination of the role of IPT for pertinent outcomes. We urge future research to fill this gap, and encourage cooperative efforts between labs to handle difficulties with recruitment, resources, and participant attrition.

### Strengths and Limitations of the Review

Apart from limitations of the reviewed studies, some limitations of the review process should be addressed. First and foremost, assessments of IPT were varying in depth and quality, and it may be argued that some studies should have been excluded for methodological reasons. However, there was no previous systematic review of the pertinent area, and imposing stricter methodological inclusion criteria would have resulted in a much more limited selection of studies (cf. Coren et al., 2018). That could, in turn, yield an overly narrow picture of the parents’ experiences. We consequently chose to include also studies with simple (though systematic) assessments of IPT. Conversely, it could also be argued that our inclusion criteria led to the exclusion of studies important for the overall picture. For instance, in a study by Booth and Booth (1993), about 60% of mothers with ID spontaneously reported experiences of IPT. This study was excluded due to unsystematic assessment of IPT. For the same reason, we excluded studies by Collings et al. (2020) and Strnadová et al. (2019), in which experiences of domestic violence were reported by 35% and 42% of the mothers, respectively. A study by Emerson and Brigham (2014), indicating that child developmental health in families headed by parents with ID is partly mediated by parental exposure to adversities (including IPT), was also excluded due to limitations in the data. Although excluded from our systematic review, these studies all add to the overall picture of IPT as a markedly common experience among parents with ID.

Notwithstanding these limitations, we reiterate that this is the first systematic review of experiences of IPT among parents with ID in the published literature. Although the studies and their findings are heterogeneous, they clearly converge on the central finding that rates of IPT are alarmingly high among parents with ID. It is imperative for future research and clinical practice to further engage with the implications of this finding.

### Concluding Remarks and Implications for Practice

We have systematically reviewed extant research with regard to IPT among parents with ID, and links between such experiences and caregiving-relevant and child developmental outcomes. Overall, we found experiences of IPT to be markedly overrepresented among parents with ID, both in unspecified and specific forms. Resonating with meta-analytic findings among parents without ID (Gardner, 2019), IPT was also consistently related to negative mental health outcomes among the parents. Results were mixed with regard to other outcomes, but a majority of the studies examining caregiving-relevant constructs indicated theoretically expectable links. Relationships between parental IPT and child development in the pertinent families remain uncertain, likely in part for methodological reasons.

On a general level, our findings provide support for the contextual-interactional model by Feldman (see Feldman & Aunos, 2020), which emphasizes that contextual risk factors, including parental experiences of IPT, influence the caregiving provided by parents with ID. Our findings also have specific implications for practice. First, practitioners should bear in mind that a substantial proportion of parents with ID have experienced IPT, and support services should include screening of such experiences in their intake routine. Second, practitioners should be aware that caregiving-relevant difficulties among parents with ID may be related to parental IPT (and other contextual factors), and adopt an openness to viewing parents’ present difficulties in the light of past and current hardships, rather than as mere consequences of their ID. Third, practitioners should be offered training in trauma-informed care, and parents suffering adverse effects of IPT should be supported in accessing suitable therapeutic interventions. When previously found wanting, it is imperative that they are offered reparative experiences by benevolent others.

## Supplemental Material

sj-pptx-1-tva-10.1177_15248380221119237 – Supplemental material for Experiences of Interpersonal Trauma Among Parents With Intellectual Disabilities: A Systematic Review Click here for additional data file. Supplemental material, sj-pptx-1-tva-10.1177_15248380221119237 for Experiences of Interpersonal Trauma Among Parents With Intellectual Disabilities: A Systematic Review by Mårten Hammarlund, Pehr Granqvist and Tommie Forslund in Trauma, Violence, & Abuse

sj-pptx-2-tva-10.1177_15248380221119237 – Supplemental material for Experiences of Interpersonal Trauma Among Parents With Intellectual Disabilities: A Systematic Review Click here for additional data file. Supplemental material, sj-pptx-2-tva-10.1177_15248380221119237 for Experiences of Interpersonal Trauma Among Parents With Intellectual Disabilities: A Systematic Review by Mårten Hammarlund, Pehr Granqvist and Tommie Forslund in Trauma, Violence, & Abuse
