# Supplementary material for: Experiences of Interpersonal Trauma Among Parents With Intellectual Disabilities: A Systematic Review
Source: Trauma Violence Abuse. 2022 Sep 5;24(4):2843–62. doi: 10.1177/15248380221119237 (PMC10486176; doi:10.1177/15248380221119237)
Supplement: sj-pptx-1-tva-10.1177_15248380221119237 – Supplemental material for Experiences of Interpersonal Trauma Among Parents With Intellectual Disabilities: A Systematic Review [file sj-pptx-1-tva-10.1177_15248380221119237.pptx]

## Slide 1
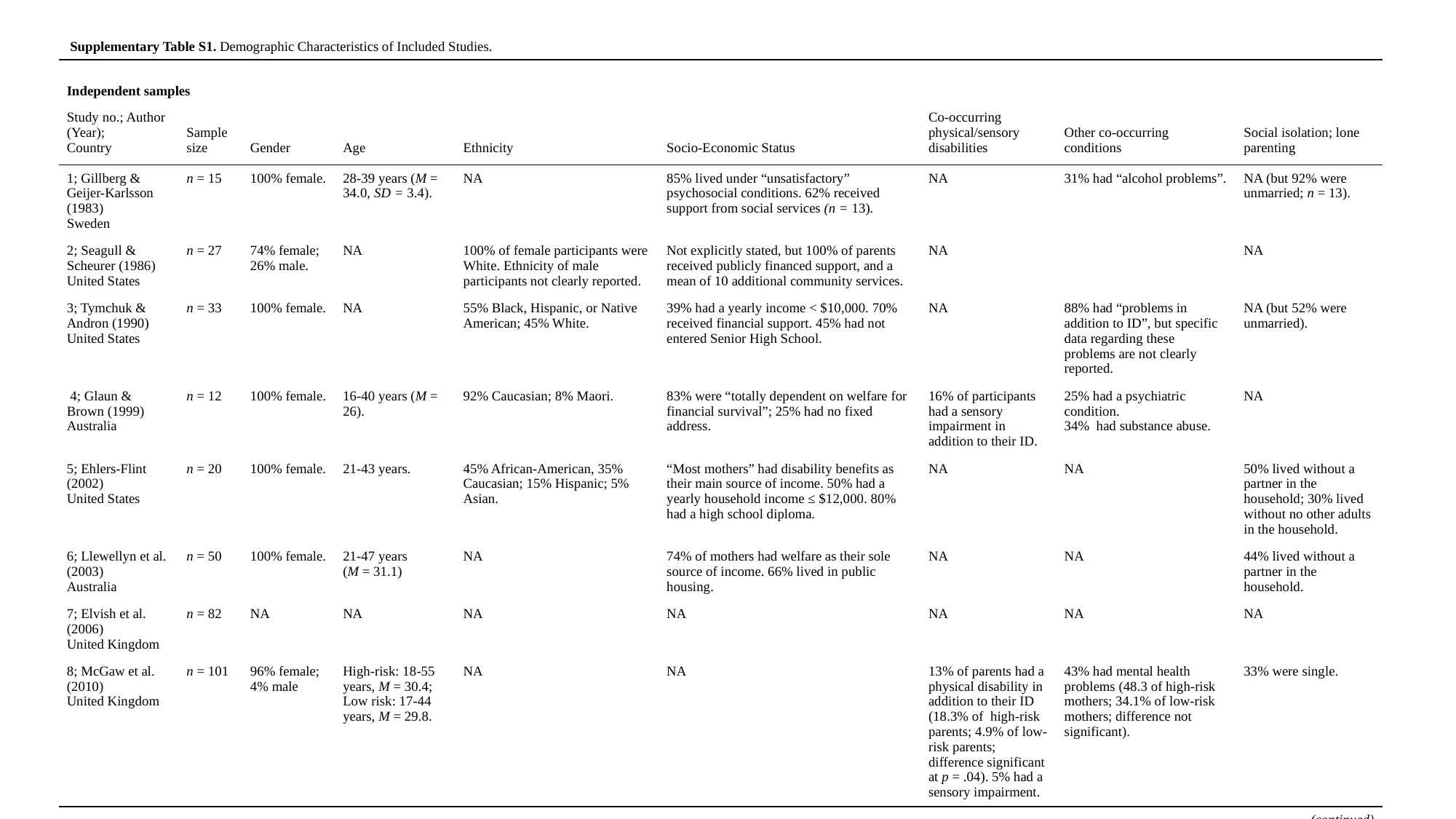

| Supplementary Table S1. Demographic Characteristics of Included Studies. | | | | | | | | |
| --- | --- | --- | --- | --- | --- | --- | --- | --- |
| Independent samples | | | | | | | | |
| Study no.; Author (Year); Country | Sample size | Gender | Age | Ethnicity | Socio-Economic Status | Co-occurring physical/sensory disabilities | Other co-occurring conditions | Social isolation; lone parenting |
| 1; Gillberg & Geijer-Karlsson (1983) Sweden | n = 15 | 100% female. | 28-39 years (M = 34.0, SD = 3.4). | NA | 85% lived under “unsatisfactory” psychosocial conditions. 62% received support from social services (n = 13). | NA | 31% had “alcohol problems”. | NA (but 92% were unmarried; n = 13). |
| 2; Seagull & Scheurer (1986) United States | n = 27 | 74% female; 26% male. | NA | 100% of female participants were White. Ethnicity of male participants not clearly reported. | Not explicitly stated, but 100% of parents received publicly financed support, and a mean of 10 additional community services. | NA | | NA |
| 3; Tymchuk & Andron (1990) United States | n = 33 | 100% female. | NA | 55% Black, Hispanic, or Native American; 45% White. | 39% had a yearly income < $10,000. 70% received financial support. 45% had not entered Senior High School. | NA | 88% had “problems in addition to ID”, but specific data regarding these problems are not clearly reported. | NA (but 52% were unmarried). |
| 4; Glaun & Brown (1999) Australia | n = 12 | 100% female. | 16-40 years (M = 26). | 92% Caucasian; 8% Maori. | 83% were “totally dependent on welfare for financial survival”; 25% had no fixed address. | 16% of participants had a sensory impairment in addition to their ID. | 25% had a psychiatric condition. 34% had substance abuse. | NA |
| 5; Ehlers-Flint (2002) United States | n = 20 | 100% female. | 21-43 years. | 45% African-American, 35% Caucasian; 15% Hispanic; 5% Asian. | “Most mothers” had disability benefits as their main source of income. 50% had a yearly household income ≤ $12,000. 80% had a high school diploma. | NA | NA | 50% lived without a partner in the household; 30% lived without no other adults in the household. |
| 6; Llewellyn et al. (2003) Australia | n = 50 | 100% female. | 21-47 years (M = 31.1) | NA | 74% of mothers had welfare as their sole source of income. 66% lived in public housing. | NA | NA | 44% lived without a partner in the household. |
| 7; Elvish et al. (2006) United Kingdom | n = 82 | NA | NA | NA | NA | NA | NA | NA |
| 8; McGaw et al. (2010) United Kingdom | n = 101 | 96% female; 4% male | High-risk: 18-55 years, M = 30.4; Low risk: 17-44 years, M = 29.8. | NA | NA | 13% of parents had a physical disability in addition to their ID (18.3% of high-risk parents; 4.9% of low-risk parents; difference significant at p = .04). 5% had a sensory impairment. | 43% had mental health problems (48.3 of high-risk mothers; 34.1% of low-risk mothers; difference not significant). | 33% were single. |
| (continued) | | | | | | | | |

## Slide 2
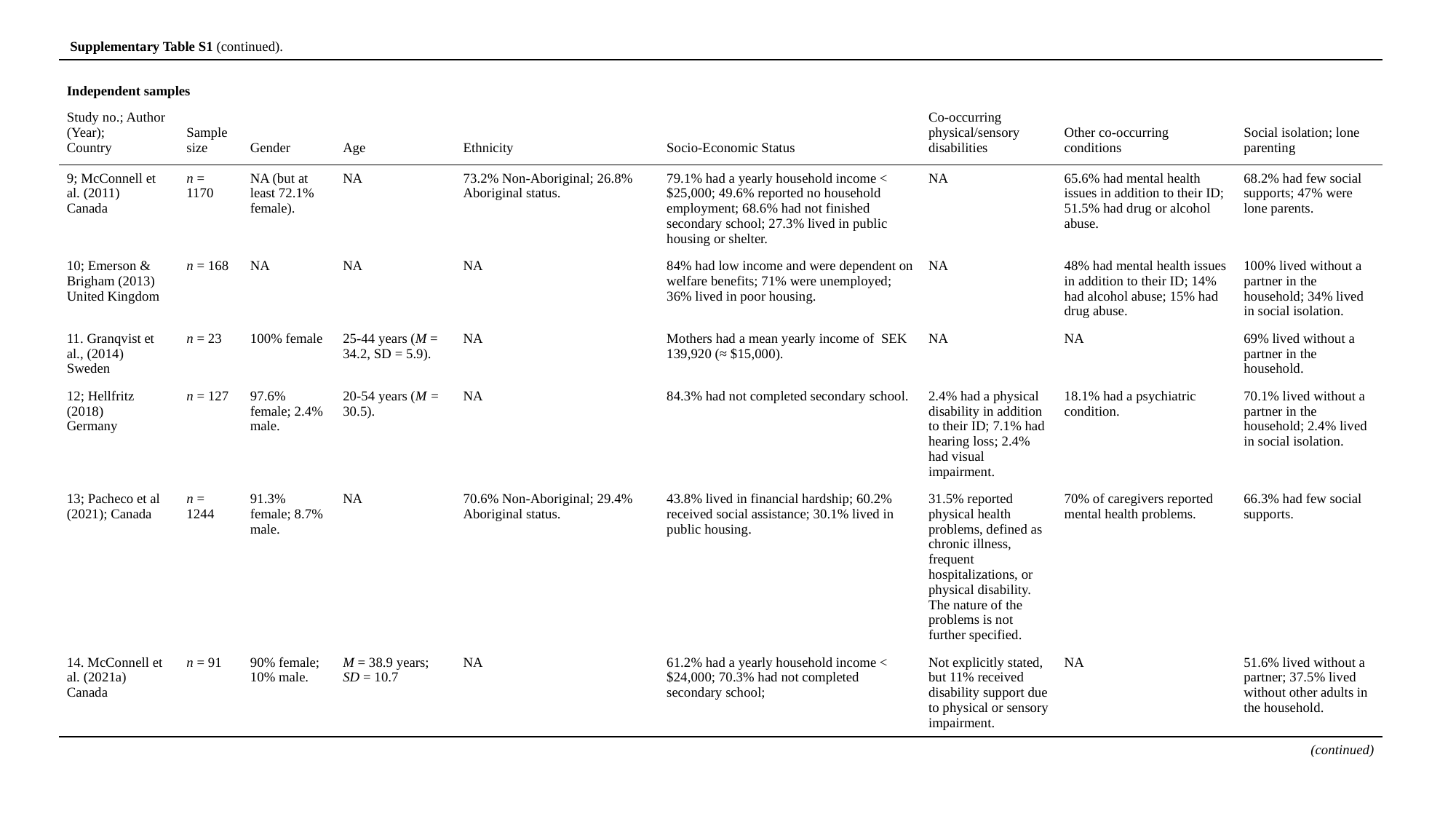

| Supplementary Table S1 (continued). | | | | | | | | |
| --- | --- | --- | --- | --- | --- | --- | --- | --- |
| Independent samples | | | | | | | | |
| Study no.; Author (Year); Country | Sample size | Gender | Age | Ethnicity | Socio-Economic Status | Co-occurring physical/sensory disabilities | Other co-occurring conditions | Social isolation; lone parenting |
| 9; McConnell et al. (2011) Canada | n = 1170 | NA (but at least 72.1% female). | NA | 73.2% Non-Aboriginal; 26.8% Aboriginal status. | 79.1% had a yearly household income < $25,000; 49.6% reported no household employment; 68.6% had not finished secondary school; 27.3% lived in public housing or shelter. | NA | 65.6% had mental health issues in addition to their ID; 51.5% had drug or alcohol abuse. | 68.2% had few social supports; 47% were lone parents. |
| 10; Emerson & Brigham (2013) United Kingdom | n = 168 | NA | NA | NA | 84% had low income and were dependent on welfare benefits; 71% were unemployed; 36% lived in poor housing. | NA | 48% had mental health issues in addition to their ID; 14% had alcohol abuse; 15% had drug abuse. | 100% lived without a partner in the household; 34% lived in social isolation. |
| 11. Granqvist et al., (2014) Sweden | n = 23 | 100% female | 25-44 years (M = 34.2, SD = 5.9). | NA | Mothers had a mean yearly income of SEK 139,920 (≈ $15,000). | NA | NA | 69% lived without a partner in the household. |
| 12; Hellfritz (2018) Germany | n = 127 | 97.6% female; 2.4% male. | 20-54 years (M = 30.5). | NA | 84.3% had not completed secondary school. | 2.4% had a physical disability in addition to their ID; 7.1% had hearing loss; 2.4% had visual impairment. | 18.1% had a psychiatric condition. | 70.1% lived without a partner in the household; 2.4% lived in social isolation. |
| 13; Pacheco et al (2021); Canada | n = 1244 | 91.3% female; 8.7% male. | NA | 70.6% Non-Aboriginal; 29.4% Aboriginal status. | 43.8% lived in financial hardship; 60.2% received social assistance; 30.1% lived in public housing. | 31.5% reported physical health problems, defined as chronic illness, frequent hospitalizations, or physical disability. The nature of the problems is not further specified. | 70% of caregivers reported mental health problems. | 66.3% had few social supports. |
| 14. McConnell et al. (2021a) Canada | n = 91 | 90% female; 10% male. | M = 38.9 years; SD = 10.7 | NA | 61.2% had a yearly household income < $24,000; 70.3% had not completed secondary school; | Not explicitly stated, but 11% received disability support due to physical or sensory impairment. | NA | 51.6% lived without a partner; 37.5% lived without other adults in the household. |
| (continued) | | | | | | | | |

## Slide 3
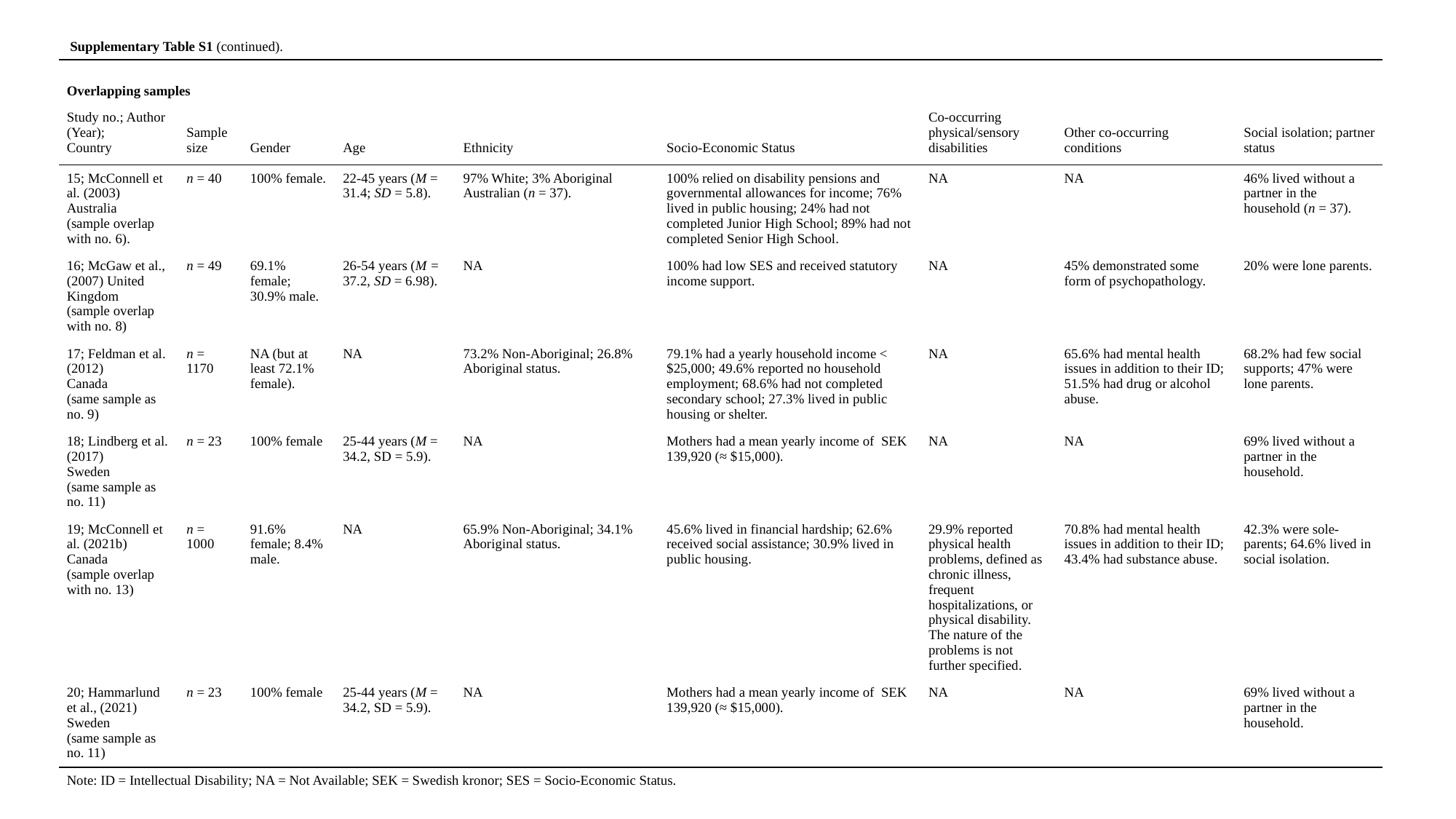

| Supplementary Table S1 (continued). | | | | | | | | |
| --- | --- | --- | --- | --- | --- | --- | --- | --- |
| Overlapping samples | | | | | | | | |
| Study no.; Author (Year); Country | Sample size | Gender | Age | Ethnicity | Socio-Economic Status | Co-occurring physical/sensory disabilities | Other co-occurring conditions | Social isolation; partner status |
| 15; McConnell et al. (2003) Australia (sample overlap with no. 6). | n = 40 | 100% female. | 22-45 years (M = 31.4; SD = 5.8). | 97% White; 3% Aboriginal Australian (n = 37). | 100% relied on disability pensions and governmental allowances for income; 76% lived in public housing; 24% had not completed Junior High School; 89% had not completed Senior High School. | NA | NA | 46% lived without a partner in the household (n = 37). |
| 16; McGaw et al., (2007) United Kingdom (sample overlap with no. 8) | n = 49 | 69.1% female; 30.9% male. | 26-54 years (M = 37.2, SD = 6.98). | NA | 100% had low SES and received statutory income support. | NA | 45% demonstrated some form of psychopathology. | 20% were lone parents. |
| 17; Feldman et al. (2012) Canada (same sample as no. 9) | n = 1170 | NA (but at least 72.1% female). | NA | 73.2% Non-Aboriginal; 26.8% Aboriginal status. | 79.1% had a yearly household income < $25,000; 49.6% reported no household employment; 68.6% had not completed secondary school; 27.3% lived in public housing or shelter. | NA | 65.6% had mental health issues in addition to their ID; 51.5% had drug or alcohol abuse. | 68.2% had few social supports; 47% were lone parents. |
| 18; Lindberg et al. (2017) Sweden (same sample as no. 11) | n = 23 | 100% female | 25-44 years (M = 34.2, SD = 5.9). | NA | Mothers had a mean yearly income of SEK 139,920 (≈ $15,000). | NA | NA | 69% lived without a partner in the household. |
| 19; McConnell et al. (2021b) Canada (sample overlap with no. 13) | n = 1000 | 91.6% female; 8.4% male. | NA | 65.9% Non-Aboriginal; 34.1% Aboriginal status. | 45.6% lived in financial hardship; 62.6% received social assistance; 30.9% lived in public housing. | 29.9% reported physical health problems, defined as chronic illness, frequent hospitalizations, or physical disability. The nature of the problems is not further specified. | 70.8% had mental health issues in addition to their ID; 43.4% had substance abuse. | 42.3% were sole-parents; 64.6% lived in social isolation. |
| 20; Hammarlund et al., (2021) Sweden (same sample as no. 11) | n = 23 | 100% female | 25-44 years (M = 34.2, SD = 5.9). | NA | Mothers had a mean yearly income of SEK 139,920 (≈ $15,000). | NA | NA | 69% lived without a partner in the household. |
| Note: ID = Intellectual Disability; NA = Not Available; SEK = Swedish kronor; SES = Socio-Economic Status. | | | | | | | | |
