# Supplementary material for: Experiences of Interpersonal Trauma Among Parents With Intellectual Disabilities: A Systematic Review
Source: Trauma Violence Abuse. 2022 Sep 5;24(4):2843–62. doi: 10.1177/15248380221119237 (PMC10486176; doi:10.1177/15248380221119237)
Supplement: sj-pptx-2-tva-10.1177_15248380221119237 – Supplemental material for Experiences of Interpersonal Trauma Among Parents With Intellectual Disabilities: A Systematic Review [file sj-pptx-2-tva-10.1177_15248380221119237.pptx]

## Slide 1
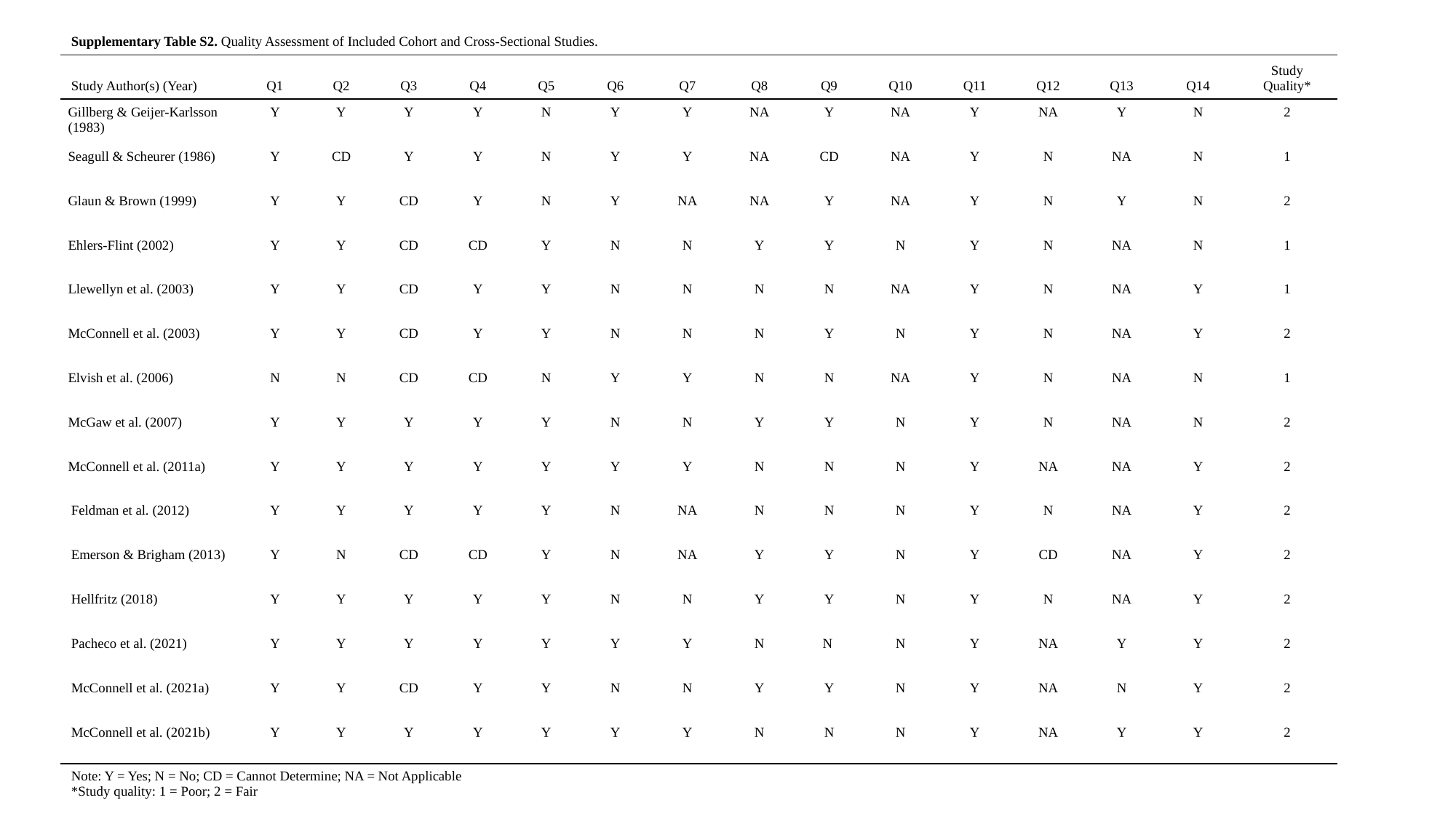

| Supplementary Table S2. Quality Assessment of Included Cohort and Cross-Sectional Studies. | | | | | | | | | | | | | | | |
| --- | --- | --- | --- | --- | --- | --- | --- | --- | --- | --- | --- | --- | --- | --- | --- |
| Study Author(s) (Year) | Q1 | Q2 | Q3 | Q4 | Q5 | Q6 | Q7 | Q8 | Q9 | Q10 | Q11 | Q12 | Q13 | Q14 | Study Quality\* |
| Gillberg & Geijer-Karlsson (1983) | Y | Y | Y | Y | N | Y | Y | NA | Y | NA | Y | NA | Y | N | 2 |
| Seagull & Scheurer (1986) | Y | CD | Y | Y | N | Y | Y | NA | CD | NA | Y | N | NA | N | 1 |
| Glaun & Brown (1999) | Y | Y | CD | Y | N | Y | NA | NA | Y | NA | Y | N | Y | N | 2 |
| Ehlers-Flint (2002) | Y | Y | CD | CD | Y | N | N | Y | Y | N | Y | N | NA | N | 1 |
| Llewellyn et al. (2003) | Y | Y | CD | Y | Y | N | N | N | N | NA | Y | N | NA | Y | 1 |
| McConnell et al. (2003) | Y | Y | CD | Y | Y | N | N | N | Y | N | Y | N | NA | Y | 2 |
| Elvish et al. (2006) | N | N | CD | CD | N | Y | Y | N | N | NA | Y | N | NA | N | 1 |
| McGaw et al. (2007) | Y | Y | Y | Y | Y | N | N | Y | Y | N | Y | N | NA | N | 2 |
| McConnell et al. (2011a) | Y | Y | Y | Y | Y | Y | Y | N | N | N | Y | NA | NA | Y | 2 |
| Feldman et al. (2012) | Y | Y | Y | Y | Y | N | NA | N | N | N | Y | N | NA | Y | 2 |
| Emerson & Brigham (2013) | Y | N | CD | CD | Y | N | NA | Y | Y | N | Y | CD | NA | Y | 2 |
| Hellfritz (2018) | Y | Y | Y | Y | Y | N | N | Y | Y | N | Y | N | NA | Y | 2 |
| Pacheco et al. (2021) | Y | Y | Y | Y | Y | Y | Y | N | N | N | Y | NA | Y | Y | 2 |
| McConnell et al. (2021a) | Y | Y | CD | Y | Y | N | N | Y | Y | N | Y | NA | N | Y | 2 |
| McConnell et al. (2021b) | Y | Y | Y | Y | Y | Y | Y | N | N | N | Y | NA | Y | Y | 2 |
| Note: Y = Yes; N = No; CD = Cannot Determine; NA = Not Applicable \*Study quality: 1 = Poor; 2 = Fair | | | | | | | | | | | | | | | |

## Slide 2
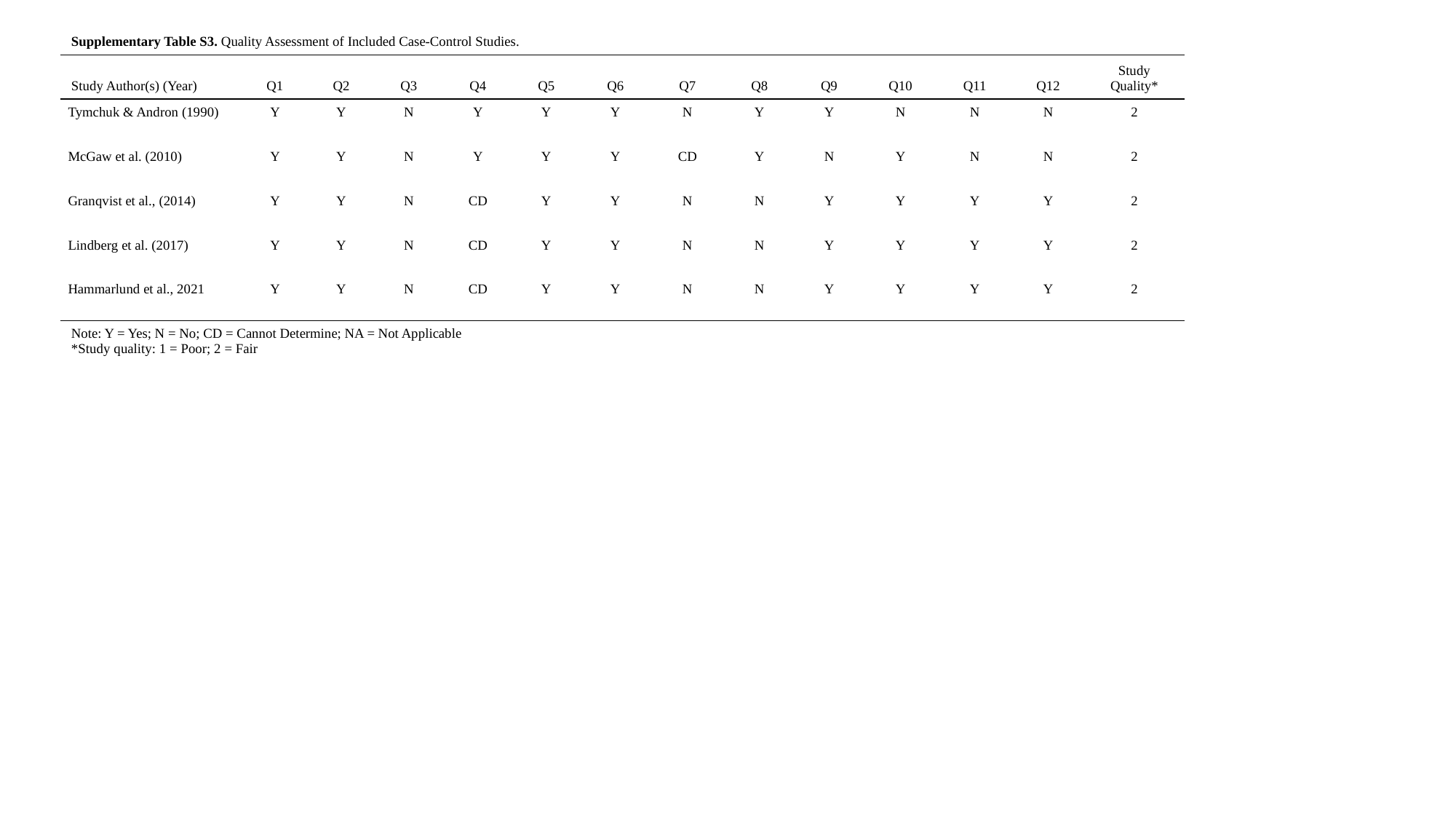

| Supplementary Table S3. Quality Assessment of Included Case-Control Studies. | | | | | | | | | | | | | |
| --- | --- | --- | --- | --- | --- | --- | --- | --- | --- | --- | --- | --- | --- |
| Study Author(s) (Year) | Q1 | Q2 | Q3 | Q4 | Q5 | Q6 | Q7 | Q8 | Q9 | Q10 | Q11 | Q12 | Study Quality\* |
| Tymchuk & Andron (1990) | Y | Y | N | Y | Y | Y | N | Y | Y | N | N | N | 2 |
| McGaw et al. (2010) | Y | Y | N | Y | Y | Y | CD | Y | N | Y | N | N | 2 |
| Granqvist et al., (2014) | Y | Y | N | CD | Y | Y | N | N | Y | Y | Y | Y | 2 |
| Lindberg et al. (2017) | Y | Y | N | CD | Y | Y | N | N | Y | Y | Y | Y | 2 |
| Hammarlund et al., 2021 | Y | Y | N | CD | Y | Y | N | N | Y | Y | Y | Y | 2 |
| Note: Y = Yes; N = No; CD = Cannot Determine; NA = Not Applicable \*Study quality: 1 = Poor; 2 = Fair | | | | | | | | | | | | | |
